# Dataset of amplicon metagenomic assessment of barley rhizosphere bacteria under different fertilization regimes

**DOI:** 10.1016/j.dib.2023.109920

**Published:** 2023-12-06

**Authors:** Olubukola Oluranti Babalola, Ben Jesuorsemwen Enagbonma

**Affiliations:** Food Security and Safety Focus Area, Faculty of Natural and Agricultural Sciences, North-West University, Private Bag X2046, Mmabatho 2735, South Africa

**Keywords:** Anthropogenic interference, Food safety, Metagenome, MG-RAST, Unclassified microbiome

## Abstract

The metagenomic dataset profiled in this research is built on bacterial 16S rRNA gene amplicon of DNA mined from barley rhizosphere under chemical (CB) and organic (OB) fertilization. Amplicon-based sequencing was prepared by the Illumina platform, and the raw sequence dataset was examined using Metagenomic Rast Server (MG-RAST). The metagenome comprised sixteen samples that include CB1 (494,583 bp), CB2 (586,532 bp), CB3 (706,685 bp), CB4 (574,606 bp), CB5 (395,460 bp), CB6 (520,822 bp), CB7 (511,729 bp), CB8 (548,074 bp), OB1 (642,794 bp), OB2 (513,767 bp), OB3 (461,293 bp), OB4 (498,241 bp), OB5 (689,497 bp), OB6 (423,436 bp), OB7 (478,657 bp) and OB8 (279,186 bp). Information from the metagenome sequences is accessible under the bioproject numbers PRJNA827679 (CB1), PRJNA827686 (CB2), PRJNA827693 (CB3), PRJNA827699 (CB4), PRJNA827706 (CB5), PRJNA827761 (CB6), PRJNA827780 (CB7), PRJNA827786 (CB8), PRJNA826806 (OB1), PRJNA826824 (OB2), PRJNA826834 (OB3), PRJNA826841 (OB4), PRJNA826853 (OB5), PRJNA827254 (OB6), PRJNA827256 (OB7), and PRJNA827257 (OB8) at NCBI. Actinobacteria dominated the soil samples at the phylum level.

Specifications Table

**Table:** 

Subject	Microbiology: rhizosphere bacteria
Specific subject area	16S rRNA gene amplicon metagenomic of barley rhizosphere bacteria under different fertilization scheme
Data format	Raw data (FastQ file)
Type of data	Table, figure and data gotten from high throughput amplicon generation sequencing
Data collection	Metagenomic DNA extraction from soil samples from barley rhizosphere under chemical fertilization (CB) and under organic fertilization (OB)*,* Next generation sequencing on Illumina (MiSeq) instrument and metagenomics classification using Ribosomal Database Project (RDP) Technology
Data source location	Institution: North-West University City/Town/Region: Madibeng, Country: North West Province Latitude and longitude (and GPS coordinates) for collected samples/data: CB = 25°39′32.2″S 27°39′49.8″E, OB = 25°39′04.9″S, 27°40′46.6″E
Data accessibility	Repository name NCBI SRA Data identification number: PRJNA827679 (CB1), PRJNA827686 (CB2), PRJNA827693 (CB3), PRJNA827699 (CB4), PRJNA827706 (CB5), PRJNA827761 (CB6), PRJNA827780 (CB7), PRJNA827786 (CB8), PRJNA826806 (OB1), PRJNA826824 (OB2), PRJNA826834 (OB3), PRJNA826841 (OB4), PRJNA826853 (OB5), PRJNA827254 (OB6), PRJNA827256 (OB7), and PRJNA827257 (OB8) Direct URL to data: https://www.ncbi.nlm.nih.gov/sra?LinkName=bioproject_sra_all&from_uid=827679 (CB1), https://www.ncbi.nlm.nih.gov/sra?LinkName=bioproject_sra_all&from_uid=827686 (CB2), https://www.ncbi.nlm.nih.gov/sra?LinkName=bioproject_sra_all&from_uid=827693 (CB3), https://www.ncbi.nlm.nih.gov/sra?LinkName=bioproject_sra_all&from_uid=827699 (CB4), https://www.ncbi.nlm.nih.gov/sra?LinkName=bioproject_sra_all&from_uid=827706 (CB5), https://www.ncbi.nlm.nih.gov/sra?LinkName=bioproject_sra_all&from_uid=827761 (CB6), https://www.ncbi.nlm.nih.gov/sra?LinkName=bioproject_sra_all&from_uid=827780 (CB7), https://www.ncbi.nlm.nih.gov/sra?LinkName=bioproject_sra_all&from_uid=827786 (CB8), https://www.ncbi.nlm.nih.gov/sra?LinkName=bioproject_sra_all&from_uid=826806 (OB1), https://www.ncbi.nlm.nih.gov/sra?LinkName=bioproject_sra_all&from_uid=826824 (OB2), https://www.ncbi.nlm.nih.gov/sra?LinkName=bioproject_sra_all&from_uid=826834 (OB3), https://www.ncbi.nlm.nih.gov/sra?LinkName=bioproject_sra_all&from_uid=826841 (OB4), https://www.ncbi.nlm.nih.gov/sra?LinkName=bioproject_sra_all&from_uid=826853 (OB5), https://www.ncbi.nlm.nih.gov/sra?LinkName=bioproject_sra_all&from_uid=827254 (OB6), https://www.ncbi.nlm.nih.gov/sra?LinkName=bioproject_sra_all&from_uid=827256 (OB7), and https://www.ncbi.nlm.nih.gov/sra?LinkName=bioproject_sra_all&from_uid=827257 (OB8)

## 1 Value of the Data

• The dataset gives understanding on the influence of different fertilizer regimes on the structure, composition and diversity of microbiome from soils obtained from barley rhizosphere.
• Microbial communities residing in soils obtained from barley rhizosphere may possibly function as a pool of biological-active molecules and unique genes necessary for biotechnological, ecological and industrial purposes.
• Barley rhizosphere emerges as one of the factors of yield potential in crops, as well as plays a significant function in improving plant fitness.
• The knowledge of the influence of conventional and organic fertilizers on the structure, composition, and diversity of the rhizosphere bacteria will assist in engineering soil to increase microbial diversity in the agroecosystem.

## 2 Data Description

**Fig. 1:**
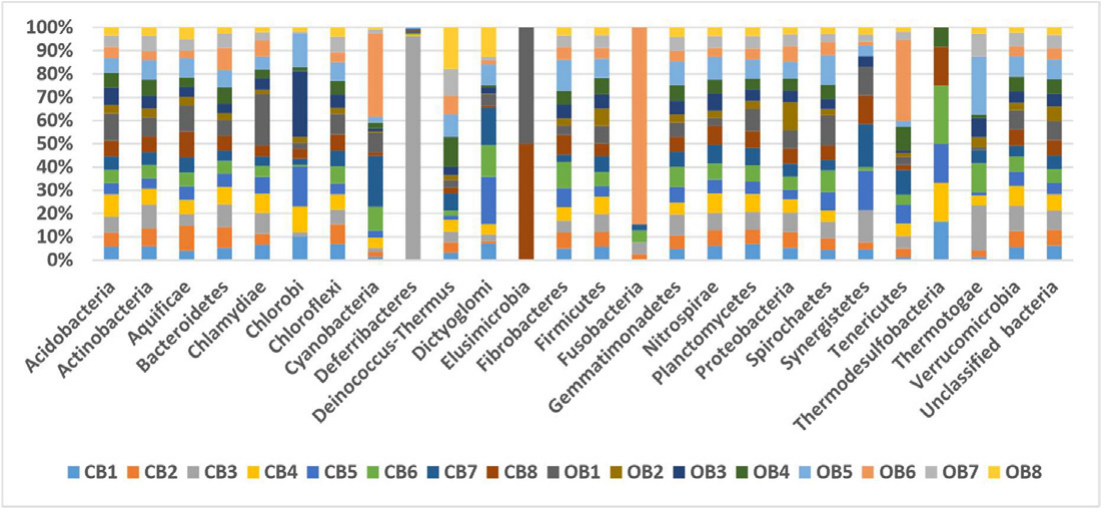
Taxonomic abundance of microbial communities among barley rhizosphere under chemical (CB) and organic (OB) fertilization.

**Fig 2:**
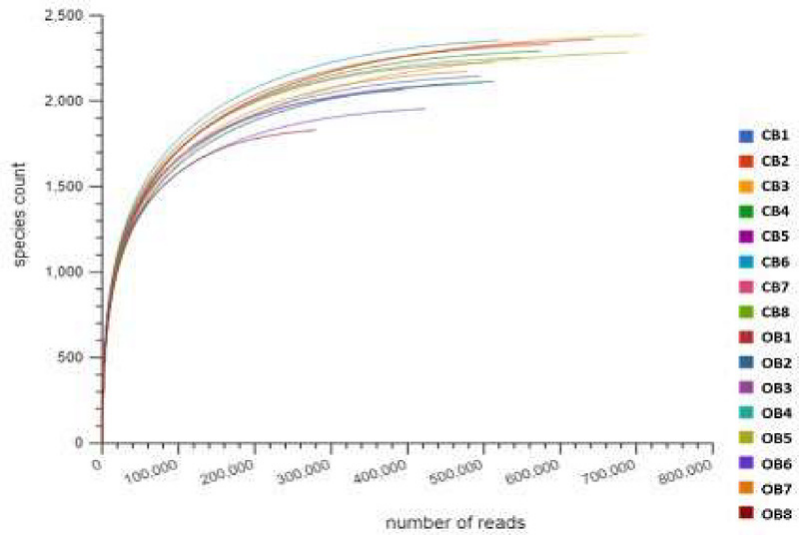
The rarefaction curves showing the species richness computation among barley rhizosphere under chemical (CB) and organic (OB) fertilization.

The metagenomic files (which is accessible under the bioproject numbers PRJNA827679 (CB1), PRJNA827686 (CB2), PRJNA827693 (CB3), PRJNA827699 (CB4), PRJNA827706 (CB5), PRJNA827761 (CB6), PRJNA827780 (CB7), PRJNA827786 (CB8), PRJNA826806 (OB1), PRJNA826824 (OB2), PRJNA826834 (OB3), PRJNA826841 (OB4), PRJNA826853 (OB5), PRJNA827254 (OB6), PRJNA827256 (OB7), and PRJNA827257 (OB8) at NCBI) comprises of raw sequences acquired via the 16S rRNA amplicon sequencing of soils from barley rhizosphere under chemical fertilization (CB1–8) and soils from barley rhizosphere under organic fertilization (OB1–8) from Madibeng, South Africa. The sequence information (Table 1), taxonomic abundance and diversity of bacterial communities of barley rhizosphere under chemical fertilization (CB) and organic fertilization (OB) are presented in Fig. 1, Fig 2 correspondingly.

**Table 1:** The sequence information of barley rhizosphere under chemical and organic fertilization.

Samples	Base count (bp)	Sequences count (bp)	Mean sequence length (bp)
CB1	144,422,171	494,583	292 ± 4
CB2	171,279,689	586,532	292 ± 3
CB3	206,379,594	706,685	292 ± 3
CB4	167,790,610	574,606	292 ± 4
CB5	115,489,933	395,460	292 ± 3
CB6	152,095,576	520,822	292 ± 4
CB7	149,445,112	511,729	292 ± 4
CB8	160,057,506	548,074	292 ± 3
OB1	187,730,488	642,794	292 ± 3
OB2	150,036,417	513,767	292 ± 2
OB3	134,731,154	461,293	292 ± 3
OB4	145,525,929	498,241	292 ± 2
OB5	201,338,767	689,497	292 ± 3
OB6	119,267,313	423,436	282 ± 49
OB7	139,777,709	478,657	292 ± 3
OB8	81,530,862	279,186	292 ± 3

CB = barley rhizosphere under chemical fertilization and OB = barley rhizosphere under organic fertilization.

## 3 Experimental Design, Materials and Methods

In this experiment, we collected sixteen soil samples of 20 g each from barley rhizosphere (the barley plants are 6 weeks old) under chemical fertilization (CB1–8) (25°39′32.2″S 27°39′49.8″E) and barley rhizosphere under organic fertilization (OB1–8) (25°39′04.9″S, 27°40′46.6″E) at a depth of 0–15 cm [1]. For the inorganic field, 75 kg ha^−1^ K_2_O, 75 kg ha^−1^ P_2_O_5_, and150 kg ha^−1^ N were the quantities of inorganic fertilizer which had been in use. Urea, potassium sulphate and calcium superphosphate were the sources of N fertilizer, K fertilizer, and P fertilizer respectively. The organic fertilizer field has been undergoing the use of cow dung of 10,625 kg ha^−1^ amount for more than a decade. The fertilization regimes employed in this study was in line with the United State Department of Agriculture (USDA, 2014). 10 × 4 m was the measurement for the respective planting field for this research and the cultivar planted on both field is barley seeds (Puma). Each of the research fields (that is CB and OB) were 10 m away. Sterile plastic bags were used to temporarily store the soil samples and then moved to cooler boxes filled with ice. On getting to the laboratory, the soil samples (after removal of debris) were stored in the refrigerator at –20 °C for further analysis. We used the Nucleospin Soil kit (Macherey-Negel, Germany) to extract the DNA of the entire barley rhizosphere microbiome by using the kit's manual as a guide.

The mined DNA was later sent to Molecular Research Laboratory, Clovis road, Texas, United States. Libraries of the 16S rRNA were arranged from the QC-passed DNA samples via the universal primers 515F (5′-GTGCCAGCMGCCGCGGTAA-3′) and 806R (5′-GGACTACHVGGGTWTCTAAT-3′) with standard Illumina barcodes and adapters. The Ampure XP beads were further used to purify the amplicons. Agilent DNA 1000 Bioanalyser was used to validate the barcoded libraries, while the quantification was done with the Qubit DNA BR reagent assay. After that, the MiSeq was used to sequence the quantified libraries that were later processed and analysed via the MG-RAST server v4.0.3 (http://metagenomics.anl.gov/) [2,3]. Raw data were uploaded as FASTQ files after demultiplexing of pairedend reads. Reads generated after quality processing and deduplication (by eliminating sequences that are: (a) greater than 5 ambiguous base pairs with 15 phred score cutoff (b) artificial sequences made by sequencing artifacts (c) have a length of greater than 2 standard deviations from the average value) by MG-RAST pipeline analysis, were subjected to annotation by using a BLAST-like alignment device called BLAT against a database that offers a nonredundant integration of numerous databases called M5NR database. Taxonomic analysis was done using RDP database under the following conditions: a maximum alignment length of 15 base pairs, an e-value of 1e^–5^ and a minimum identity of 60%. MG-RAST pipeline made available estimation of microbial abundances present in barley rhizosphere under chemical fertilization (CB) and barley rhizosphere under organic fertilization (OB).

After the MG-RAST analysis on the sequences, we visualized them by plugging them with a stacked bar chart in Excel (version 2010) to appraise the taxonomic abundance. An evaluation of the species richness was done with rarefaction curves from the MG-RAST tool.

## Limitations

Not applicable.

## Ethics Statement

The current work follows the ethical requirements for publication in Data in Brief. It does not involve human subjects, animal experiments, or any data collected from social media platforms.

## CRediT authorship contribution statement

**Olubukola Oluranti Babalola:** Conceptualization, Supervision, Project administration, Resources. **Ben Jesuorsemwen Enagbonma:** Writing – original draft, Writing – review & editing, Formal analysis, Data curation, Investigation.

## Data Availability

Barley rhizosphere microbiome under different fertilization scheme (Original data) (NCBI) Barley rhizosphere microbiome under different fertilization scheme (Original data) (NCBI) Barley rhizosphere microbiome under different fertilization scheme (Original data) (NCBI) Barley rhizosphere microbiome under different fertilization scheme (Original data) (NCBI) Barley rhizosphere microbiome under different fertilization scheme (Original data) (NCBI) Barley rhizosphere microbiome under different fertilization scheme (Original data) (NCBI) Barley rhizosphere microbiome under different fertilization scheme (Original data) (NCBI) Barley rhizosphere microbiome under different fertilization scheme (Original data) (NCBI) Barley rhizosphere microbiome under different fertilization scheme (Original data) (NCBI) Barley rhizosphere microbiome under different fertilization scheme (Original data) (NCBI) Barley rhizosphere microbiome under different fertilization scheme (Original data) (NCBI) Barley rhizosphere microbiome under different fertilization scheme (Original data) (NCBI) Barley rhizosphere microbiome under different fertilization scheme (Original data) (NCBI) Barley rhizosphere microbiome under different fertilization scheme (Original data) (NCBI) Barley rhizosphere microbiome under different fertilization scheme (Original data) (NCBI) Barley rhizosphere microbiome under different fertilization scheme (Original data) (NCBI)
